# Improved Production Efficiency of Virus-Like Particles by the Baculovirus Expression Vector System

**DOI:** 10.1371/journal.pone.0140039

**Published:** 2015-10-12

**Authors:** Javier López-Vidal, Silvia Gómez-Sebastián, Juan Bárcena, Maria del Carmen Nuñez, Diego Martínez-Alonso, Benoit Dudognon, Eva Guijarro, José M. Escribano

**Affiliations:** 1 Alternative Gene Expression S.L. (ALGENEX), Edificio de empresas, Campus Montegancedo (Universidad Politécnica de Madrid), Pozuelo de Alarcón, Madrid, Spain; 2 Centro de Investigación en Sanidad Animal (CISA-INIA), Valdeolmos, Madrid, Spain; 3 Departamento de Biotecnología, Instituto Nacional de Investigación y Tecnología Agraria y Alimentaria (INIA), Autovia A6 Km 7, Madrid, Spain; Sun Yat-sen University, CHINA

## Abstract

Vaccines based on virus-like particles (VLPs) have proven effective in humans and animals. In this regard, the baculovirus expression vector system (BEVS) is one of the technologies of choice to generate such highly immunogenic vaccines. The extended use of these vaccines for human and animal populations is constrained because of high production costs, therefore a significant improvement in productivity is crucial to ensure their commercial viability. Here we describe the use of the previously described baculovirus expression cassette, called TB, to model the production of two VLP-forming vaccine antigens in insect cells. Capsid proteins from porcine circovirus type 2 (PCV2 Cap) and from the calicivirus that causes rabbit hemorrhagic disease (RHDV VP60) were expressed in insect cells using baculoviruses genetically engineered with the TB expression cassette. Productivity was compared to that obtained using standard counterpart vectors expressing the same proteins under the control of the *polyhedrin* promoter. Our results demonstrate that the use of the TB expression cassette increased the production yields of these vaccine antigens by around 300% with respect to the standard vectors. The recombinant proteins produced by TB-modified vectors were fully functional, forming VLPs identical in size and shape to those generated by the standard baculoviruses, as determined by electron microscopy analysis. The use of the TB expression cassette implies a simple modification of the baculovirus vectors that significantly improves the cost efficiency of VLP-based vaccine production, thereby facilitating the commercial viability and broad application of these vaccines for human and animal health.

## Introduction

Recent decades have witnessed the growing use of baculovirus vectors to produce and obtain a range of recombinant proteins [1,2]. The production system most commonly used is based on *Autographa californica* multinuclear polyhedrosis virus (AcMNPV). Since the development of the baculovirus expression vector system (BEVS) in the ‘80s [3], thousands of recombinant proteins, ranging from cytosolic enzymes to membrane-bound proteins, have been successfully produced in baculovirus-infected insect cells.

Recently, a baculovirus vector expression cassette containing rearranged baculovirus-derived genetic regulatory elements acting in cascade has been described [4]. This novel cassette, denominated TB, conferred significant production improvements to the BEVS, including prolonged cell integrity after infection, improved protein integrity, and up to a 4-fold increase in the production yield of a recombinant model protein (green fluorescent protein) with respect to a standard baculovirus vector. The expression cassette consists of a cDNA encoding for the baculovirus transactivation factors IE1 and IE0, expressed under the control of the *polyhedrin* (*polh*) promoter, and a homologous repeated transcription enhancer sequence operatively *cis*-linked to *p10* chimeric promoters.

Virus-like particles (VLPs) present viral antigens in a more authentic conformation than monomeric structural proteins. VLPs mimic the structure of virus particles they are derived from and display excellent adjuvant properties, being capable of inducing innate and cognate immune responses [5]. In this regard, VLPs present high-density B-cell epitopes for antibody production and intracellular T-cell epitopes, thus inducing potent humoral and cellular immune responses, respectively [6,7]. Therefore, recombinant VLPs are more preferable for vaccine and diagnostic purposes. Some VLP-based vaccines have been licensed and commercialized, others have entered clinical development, while many are in the proof-of-concept stage [8,9]. The BEVS has been widely used to produce these vaccines [10–13].

Circoviruses belong to the *Circoviridae* family and infect a variety of animal and plant species. They are named after their circular single-stranded DNA (ssDNA) genome, and they represent the smallest DNA viruses infecting mammals. Porcine circovirus 2 (PCV2) causes considerable mortality among pigs and is a serious problem for the swine industry worldwide, leading to a significant drop in the profitability of pork production [14]. PCV2 is a non-enveloped virus with a genome of approximately 1,767 bases packed in an icosahedral virion particle with a diameter of between 12 and 23 nm. A single structural protein of the viral coat encoded by ORF2 on the complementary strand is the only structural capsid (Cap) protein [15]. Expression of PCV2 Cap in insect cells results in recombinant protein self-assembly into VLPs that are structurally and antigenically indistinguishable from regular PCV2 capsids [16]. These VLPs comprise two of the subunit vaccines currently on the market (Porcilis^®^ PCV and CircoFLEX^®^).

Rabbit hemorrhagic disease (RHD) is a highly contagious and lethal infection that affects both wild and domestic rabbits (*Oryctolagus cuniculus*). It is a major threat to wildlife and to trade in rabbit-derived products (reviewed in [17]). Its etiological agent, rabbit hemorrhagic disease virus (RHDV), is a small non-enveloped virus, a prototype of the *Lagovirus* genus included in the *Caliciviridae* family [18,19]. RHDV particles consist of a 7.5-kb single-stranded positive sense RNA genome in a small icosahedrical capsid of 38 nm in diameter. The RHDV coat protein (VP60) has an apparent molecular weight of 60 kDa. One hundred and eighty copies of this protein are assembled to produce native virus capsids and VLPs [20]. Typically, RHD is resolved within 48 h to 72 h post-infection (hpi) and it is fatal in 80% of adult animals, causing acute liver damage and disseminated intravascular coagulation [5,21].

The lack of an efficient *in vitro* propagation system for RHDV has hindered the large-scale production of the virus as source of vaccine antigens. Vaccines are currently produced by the chemical inactivation of crude virus preparations obtained from the livers of infected rabbits [9]. Several heterologous systems, including insect cells and insect larvae, have been used to produce recombinant versions of VP60 using recombinant baculoviruses [17,22–26]. In 2010, a new RHDV variant (RHDVb) was described. This variant has virtually replaced the previous circulating RHDV strain [27,28]. Vaccines against genogroup 1 viruses are not protective against this new variant.

The purpose of this study is to demonstrate the efficiency of TB-modified baculoviruses to produce VLPs using as models the PCV2 and RHDV capsid proteins.

## Methods and Materials

### Cell culture and viruses

*Spodoptera frugiperda* (*Sf*21 and *Sf*9) cell lines were cultured at 27°C in TNMFH medium (PAN Biotech GmbH, Germany) with 10% heat-inactivated fetal bovine serum (PAN Biotech GmbH) and gentamicin (50 μg/ml) (PAN Biotech GmbH). Cell density and viability were assessed by Trypan blue staining. Cell viability was calculated on the basis of the percentage of living cells with respect to the total number of cells at various times post-infection.

The *Sf9* cells, which were cultured in suspension, were infected in spinner flasks (80 ml of culture media) at a cell density of 2x10^6^ cells/ml. Cell viability at the time of infection was >95% in monolayer and >99% in suspension.

### Plasmid constructions and recombinant baculovirus generation

The TB plasmid, pFBTBVP1, was constructed by cloning the nucleotide sequence of the gene encoding for the VP1+VP2 proteins from a RHDV genogroup 1 (AST89; GenBank reference Z49271). The sequence (flanked by XhoI/XbaI) was PCR-amplified from a previous vector, pHph306GS [21], using the primers VP60F (5´-CTCGAGATAAATATGGAGGGCAAA3´) and VP60R (5 [ 7 ]´-TCTAGAATAGCTTACTTTAAACTATA-3´), and subcloned into the pFBTB vector [4] to obtain pFBTBVP1. To generate pFBVP1, the VP1+VP2 encoding sequence was extracted from the pHph306GS vector using the restriction enzymes *BamH*I and *Kpn*I and subcloned into the pFastBac1.

**Fig 1:**
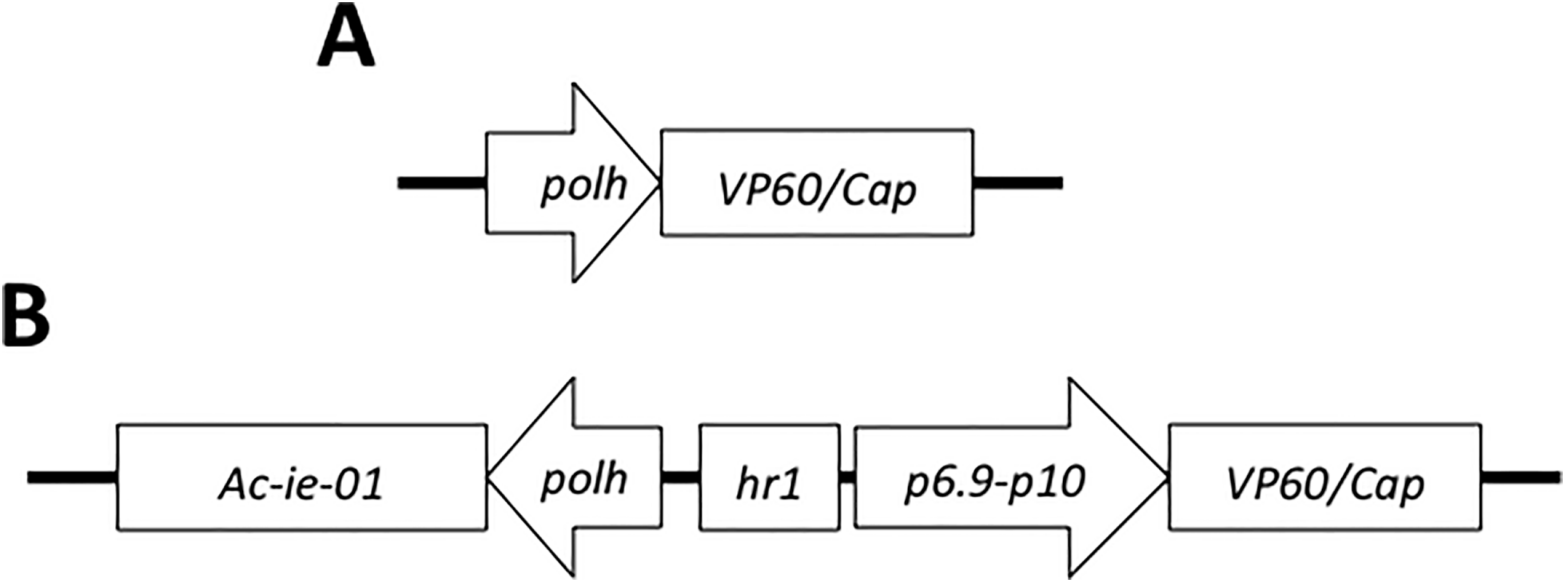
Schematic representation of the two constructs used to generate recombinant baculoviruses expressing the recombinant proteins RHDV-VP60 and PCV2-Cap. A) Expression cassette used to express the recombinant proteins under the control of the *polh* promoter in a conventional TB(-) baculovirus. B) Expression cassette used to express the recombinant proteins in a TB(+) baculovirus containing all genetic regulatory elements, including the transactivation elements encoded by *Ac-ie-01* sequence, the enhancer sequence *hr1*, and the combination of promoters *p6*.*9* and *p10*.

The pFBTBCap transfer vector was constructed by cloning the *ORF2* sequence encoding for the porcine circovirus Cap protein from a PCV2a strain (GER3; GenBank reference AF201307), contained in a previously generated pFastBac transfer vector (34). The sequence was amplified by PCR using the primers pCapXhoI F (5´-CTCGAGATGACGTATCCAAGGAGG-3´) and pCapNcoI R (5´-CCATGGTTAGGGTTTAAGTGGGGGG-3´), which include the *Xho*I and *Nco*I restriction sites flanking the coding sequence. The *ORF2* sequence was then subcloned into the pFBTB donor vector. The resulting transfer vectors are summarized in Fig 1.

The recombinant baculoviruses (rBacs) were obtained by generating the bacmids by means of the pFastBac1 vector for use with the Bac-To-Bac baculovirus expression system (Invitrogen, Life Technologies). The TB vectors were constructed by cloning the TB cassette into the pFastBac vectors, as described previously [4]. Bacmids were transfected into *Sf*21 cells using Cellfectin^®^II Reagent (Invitrogen, Life Technologies) and following the manufacturer's instructions. The resulting rBacs, TB(-) or TB(+), were then passaged twice and titrated in duplicate by plaque assays in 6-well plates in *Sf21* cells. Virus titers were expressed as plaque-forming units (pfu).

### Infection of *Sf9* insect cells

*Sf*9 cells were infected *in vitro* with recombinant baculoviruses at a multiplicity of infection (MOI) of 0.1. Cells were monitored to check for viability by Trypan blue staining, harvested at several hours post-infection (hpi), and then frozen until processing.

### Analysis of protein extracts

*Sf*9 cells infected with the rBacs at a MOI of 0.1 were harvested and sedimented by centrifugation. The supernatants were removed, and the pellets were resuspended in PBS pH 7.2 and disrupted by three cycles of freezing (-196°C) and thawing (37°C). Cellular debris was removed by centrifugation at 23,660 *× g* for 5 min at 4°C. Protein concentrations were determined by the Bradford method using a protein assay kit (Bio Rad Laboratories). Thirty micrograms of total soluble protein (TSP) fractions from infected cells were resolved in 15% SDS-PAGE gels for PCV2-Cap and 8% SDS-PAGE gels for RHDV-VP60. Gels were stained with Coomassie blue or transferred to nitrocellulose membranes. Immune detection of Cap and VP60 (encoded by VP1 gene) was performed by Western blot. The analysis was done using a monoclonal antibody against the PCV2-Cap protein (Ab 36A9, Ingenasa) diluted 1:1000 or a monoclonal antibody against the RHDV-VP60 protein (anti-RHDV, Ingenasa) diluted 1:1000, followed by incubation with an anti-IgG mouse horseradish peroxidase (HRP) labelled (GE Healthcare) diluted 1:2000.

Soluble recombinant Cap and VP60 proteins produced in *Sf9* cells was measured by band densitometry with the ChemiDoc™ XRS Gel Imaging System using Image Lab^TM^ software (Bio-Rad^TM^). A BSA standard curve was used for quantification.

### VLP purification

The VLPs were extracted from infected *Sf9* cells at 96 hpi by centrifugation in the presence of detergents essentially as described [29]. Briefly, *Sf9*-infected cells at 96 hpi were sedimented by centrifugation (700 x *g* 5 min at 4°C). The pellet was washed twice in PBS 1X and resuspended in distilled H_2_O with protease inhibitors (Complete^®^, Roche) for cell lysis and then treated with DNAse I (Roche Diagnostics) for 1 h at 37°C. Later, 2% sarkosyl (Sigma) and 5 mM EDTA (Sigma) in a PBSv (0.2 M sodium phosphate, 0.1 M NaCl, pH 6.0) were added, and samples were incubated overnight at 4°C. After an additional centrifugation (2,000 x *g*, 5 min), supernatants were subjected to ultracentrifugation (131,453 x *g*; 2.5 h). Sediments were extracted twice in Vertrel (Sigma) and submitted to second ultracentrifugation (131,453 x *g*; 2.5 h). Finally, sediments were resuspended in 1ml of PBS and stored at 4°C until analysis.

### Electron microscopy analysis

Electron microscopy analyses were performed by conventional means. Briefly, purified VLPs (approximately 5 μl) were applied to glow-discharged carbon-coated grids for 2 min. Samples were negatively stained with 2% (w/v) aqueous uranyl acetate. Micrographs were recorded with an EM 2000 Ex microscope (JEOL, Japan).

## Results

### Production of VLPs from PCV2-Cap antigen by a TB-modified baculovirus

Two baculoviruses expressing the ORF2 (PCV2-Cap) gene under the control of the *polh* promoter (TB(-)) (Fig 1A), or in the context of the TB expression cassette (Fig 1B) (TB(+)) were constructed and used to infect *Sf9* cells in suspension at a MOI of 0.1. The expression of the PCV2-Cap antigen induced by the two baculoviruses was detected in SDS-PAGE and confirmed by Western blot using a specific primary mouse monoclonal antibody.

**Fig 2:**
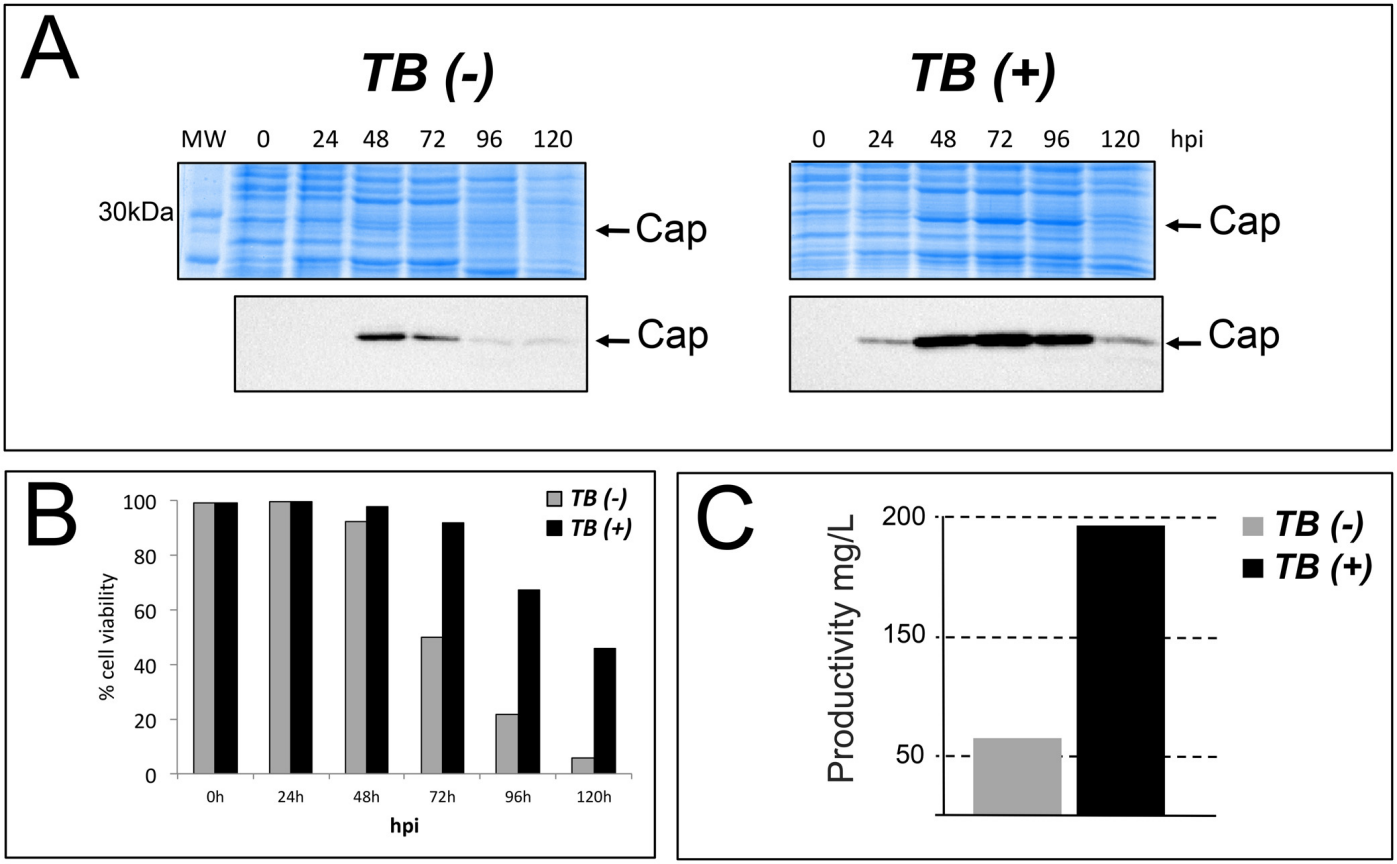
Production of the PCV2-Cap protein in *Sf9* cells grown in suspension and infected by a conventional TB(-) or by a TB (+) baculovirus expressing the ORF2 from porcine circovirus gene. A) Coomassie blue staining of SDS-PAGE gels resolving protein extracts obtained from cells infected with TB(-) or TB(+) baculoviruses and collected from 0 to 120 hpi. The same samples were submitted to a Western blot using a monoclonal antibody against the PCV2-Cap antigen. B) Infected cells viability at different hpi with TB(-) and TB(+) baculoviruses expressing the PCV2-Cap protein, analyzed by Trypan blue staining. This panel represents the mean values of three different experiments. C) Calculation of PCV2-Cap protein productivity in *Sf9* cells infected by TB(-) and TB(+) baculoviruses at the optimal production time in each case (24 hpi for TB(-) and 48 hpi for the TB(+) baculovirus).

The expression of PCV2-Cap protein induced by the TB(-) baculovirus was initially detected by Coomassie blue staining at 48 hpi and presented a peak of maximum productivity at this time, as also determined by Western blot (Fig 2A). When this antigen was expressed by the TB(+) baculovirus, the recombinant protein was initially detected at 24 hpi, presenting a peak of productivity at 72 hpi (Fig 2A). The TB(+) baculovirus efficiently expressed the PCV2-Cap protein up to at least 96 hpi. In contrast, the TB(-) baculovirus showed a dramatic decrease in protein yield after 72 hpi (Fig 2A). Cells infected by the TB(+) baculovirus showed an increase in cell viability with respect to cells infected by the control vector after 72 hpi (Fig 2B).

Quantification of PCV2-Cap production yields of the two baculoviruses at the maximum peak of productivity (48 and 72 hpi for TB(-) and TB(+) respectively) showed differences of more than 300%. While the standard TB(-) baculovirus reached a yield of around 56 mg/L, the TB(+) baculovirus produced around 198 mg/L (Fig 2C). Two additional repetitive PCV2-Cap expression analyses in *Sf9* cells using the same infection conditions showed identical relative production yields among the TB(-) and TB (+) baculoviruses.

### Production of VLPs from RHDV-VP60 antigen by a TB-modified baculovirus

As in the case of the PCV2-Cap protein, two baculovirus vectors (TB(-) and TB(+) (Fig 1) were constructed and used to infect *Sf9* cells in suspension at a MOI of 0.1. The expression of the RHDV-VP60 antigen induced by the two baculoviruses was detected in SDS-PAGE by Coomassie blue staining and confirmed by Western blot using a primary mouse monoclonal antibody against the VP60 protein.

**Fig 3:**
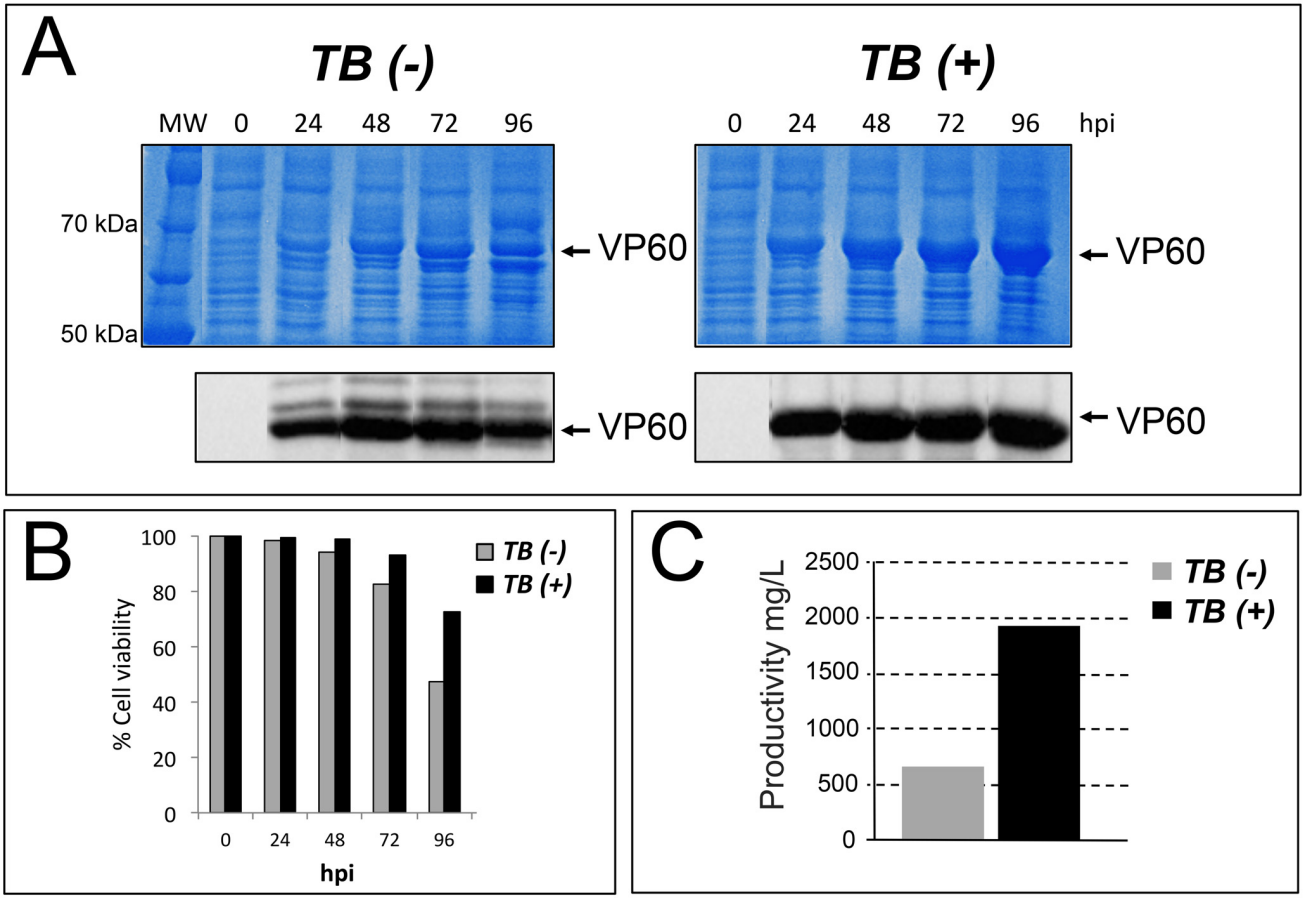
Production of the RHDV-VP60 protein in *Sf9* cells grown in suspension and infected by a conventional TB(-) or by a TB (+) baculovirus expressing the VP1 and VP2 genes from RHDV genogroup 1. A) Coomassie blue staining of SDS-PAGE gels resolving protein extracts obtained from infected cells with TB(-) or TB(+) baculoviruses and collected from 0 to 96 hpi. The same samples were submitted to a Western blot using a monoclonal antibody against the RHDV-VP60 antigen. B) Infected cells viability at different hpi with TB(-) and TB(+) baculoviruses expressing the RHDV-VP60 protein, analyzed by Trypan blue staining. This panel represents the mean values of three different experiments. C) Calculation of VP60 protein productivity in *Sf9* cells infected by TB(-) and TB(+) baculoviruses at the optimal production time in each case (72 hpi for TB(-) and 96 hpi for the TB(+) baculovirus).

The expression of RHDV-VP60 protein induced by the TB(-) baculovirus was detected by Coomassie blue staining after 24 hpi, presenting a peak of maximum productivity at 48–72 hpi, as also determined by Western blot (Fig 3A). When this antigen was expressed by the TB(+) baculovirus, the protein was also initially detected at 24 hpi but to a greater extent, presenting a peak of productivity at 96 hpi (Fig 3A). In contrast to the TB(+) baculovirus, at this time point the TB(-) baculovirus showed a significant decreased in RHDV-VP60 yield (Fig 3A). Interestingly, extracts from cells infected by the TB(+) baculovirus showed a single reactive band by Western blot, while similar extracts obtained after infection with the TB(-) baculovirus showed minor reactive bands of distinct molecular weights, thereby suggesting the presence of aberrant forms of the protein (Fig 3A). Cells infected by the TB(+) baculovirus showed an increase in cell viability with respect to cells infected by the control vector after 72 hpi (Fig 3B).

Quantification of RHDV-VP60 production yields by the two baculoviruses at the maximum peak of productivity (72 and 96 hpi for TB(-) and TB(+) respectively) showed differences of around 290%. While the standard baculovirus reached a yield of around 0.65g/L, the TB(+) baculovirus produced around 1.9g/L (Fig 3C). Two additional repetitive expression analyses of RHDV-VP60 in *Sf9* cells using the same infection conditions showed identical relative production yields among the TB(-) and TB (+) baculoviruses.

### Electron microscopy characterization of PCV2 and RHDV VLPs produced in insect cells by the TB-modified baculoviruses

**Fig 4:**
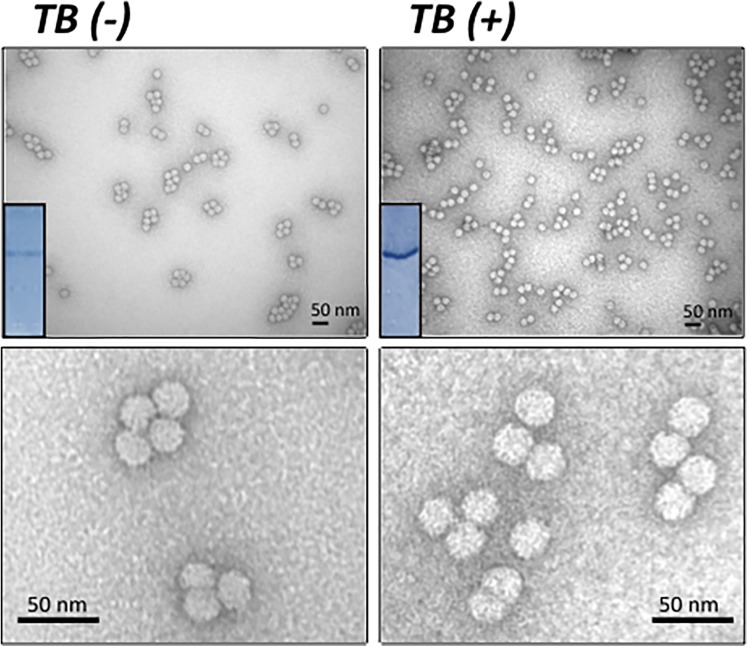
VLPs formed after infection of *Sf9* cells with a conventional TB(-) or a TB (+) baculovirus expressing the ORF2 from porcine circovirus. Extracts from infected cells at the optimal production times with each baculovirus were processed for VLP purification. The protein patterns of the purified VLPs obtained with each baculovirus are shown in the inserts (Coomassie blue stained). Samples were observed by Electron microscopy using negative staining. The figure shows the VLPs at two magnifications. VLPs obtained with the two baculoviruses presented identical sizes and shapes but were more abundant when produced by the TB(+) virus. The micrographs are representative of the fields analyzed.

**Fig 5:**
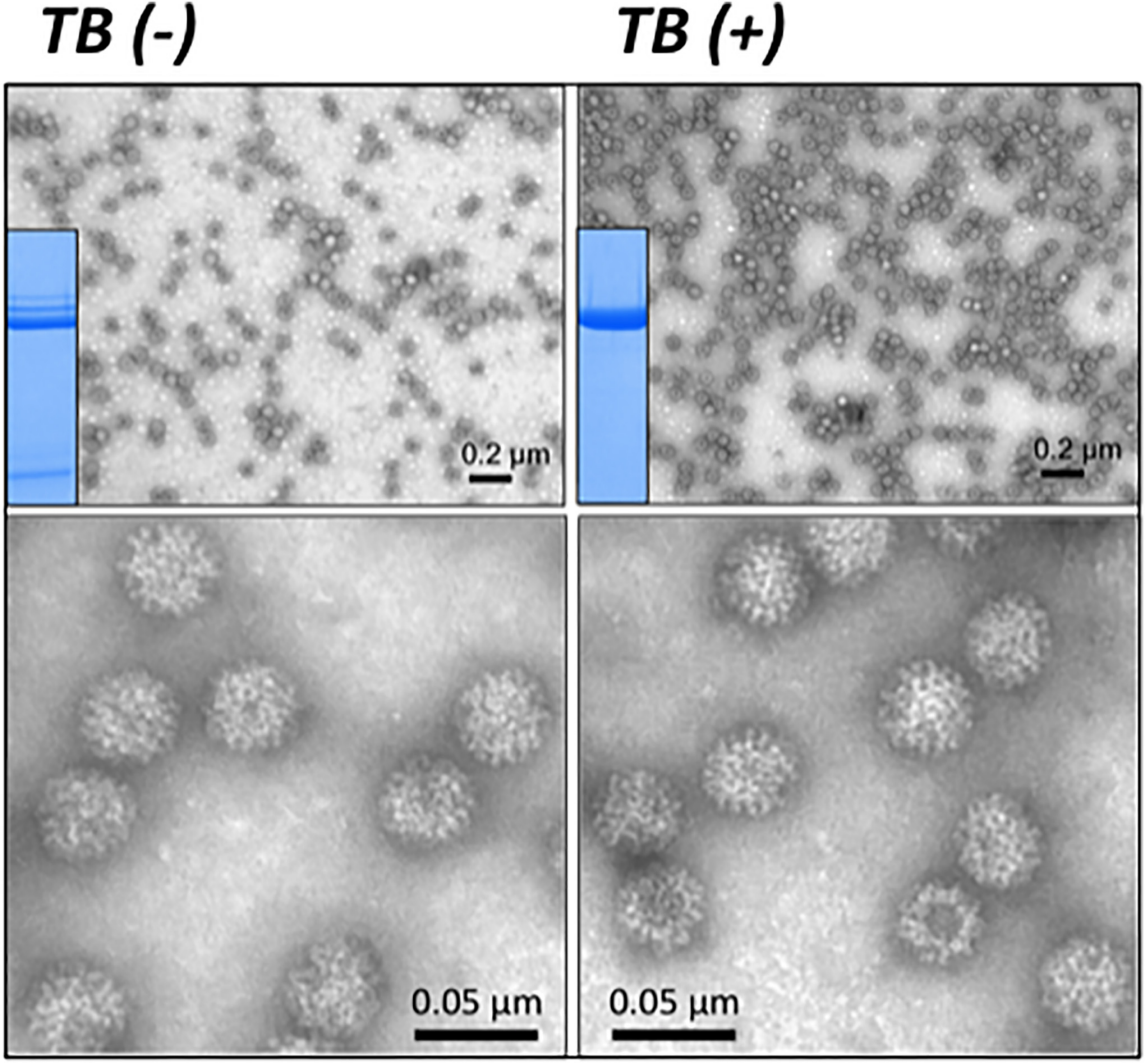
VLPs formed after infection of Sf9 cells with a conventional TB(-) or a TB (+) baculovirus expressing the VP1 and VP2 encoding genes from RHDV. Extracts from infected cells at the optimal production times with each baculovirus were processed for VLP purification. The protein patterns of purified VLPs obtained with each baculovirus are shown in the inserts (Coomassie blue stained). Samples were observed by Electron microscopy using negative staining. The figure shows the VLPs at two magnifications. VLPs obtained with the two baculoviruses presented identical sizes and shapes but were more abundant when produced by the TB(+) virus. The micrographs are representative of the fields analyzed.

All recombinant PCV2-Cap and RHDV-VP60 proteins were found in the soluble fraction of cell lysates, thus allowing their efficient purification. The PCV2-Cap- and RHDV-VP60-based VLPs generated in infected insect cells were purified by ultracentrifugation and examined using electron microscopy (EM). Despite the differences in expression levels induced by the TB(-) and TB(+) baculoviruses, all four purified PCV2-Cap- and RHDV-VP60-based VLPs assembled into regular particles that were homogenous in size and shape, similar to native PCV2 and RHDV virions (Figs 4 and 5). However, inspection of various preparations revealed significant differences in the densities of VLPs among preparations obtained with the TB(-) and TB(+) baculoviruses when the purification process started with the same number of infected cells (Figs 4 and 5). The higher PCV2-Cap and RHDV-VP60 yields mediated by TB(+) baculoviruses correlated with higher densities of VLPs observed by EM.

## Discussion

Insect cells in combination with baculovirus vectors are currently used to produce at least seven commercial vaccines, and many other vaccines are at different clinical phase stages using this expression technology. The self-assembled nanostructures called VLPs hold tremendous potential as vaccine candidates since they preserve the repetitive ordered arrangement of epitopes on their surfaces [30,31]. VLPs mimic the three-dimensional nature of viruses while lacking the genome packaged inside their capsid. VLPs therefore provide a safe and effective approach for inducing neutralizing antibodies against surface proteins, where soluble forms of their protein subunits have failed. VLPs can also be exploited as ‘‘platforms” for the presentation of foreign epitopes and/or targeting molecules on chimeric VLPs [32]. VLP chimeras have been extensively explored as vaccine candidates over the last 20 years [33]. With the advent of successful cases of approved VLP-based vaccines (3 out of the 7 marketed subunit vaccines produced in insect cells), pharmaceutical companies are now redirecting resources to the development of such products. However, a VLP-based product candidate cannot be competitive in the market unless its manufacturing process is scalable and cost-effective [34]. The BEVS is an expression system of choice for these purposes. In this regard, although insect cells yield some differences in the glycosylation pattern of the final products, BEVS is highly versatile and efficiently produces structurally and immunologically functional VLPs.

Insect cells are perfectly adapted to suspension conditions, thereby allowing for large-scale upstream processes. Furthermore, pharmaceutical companies have improved many aspects related to cell density and media optimization to achieve greater production yields. However, insect cells infected by recombinant baculoviruses rarely exceed production yields of 50 to 100 mg/L [32]. This value is far from the yields achieved by other production platforms, which provide g/L for other proteins of lower complexity. However, yield is not the only bottleneck of the BEVS. A marked proteolysis of recombinant proteins during baculovirus-based production is frequently encountered. This process is due, in part, to the cytopathogenic effects of the baculovirus vectors in insect cells during infection [35–37].

Considerable research effort has been channeled into increasing the productivity of the BEVS. Some strategies include maltose binding protein, glutathione S transferase [38], SUMO [39] and KDEL retention signal [40]. The deletion of other non-essential virus genes encoding for proteins such as p26, p10 and p74 also offer advantages for productivity [41]. Other attempts to improve the stability of the proteins expressed have focused on two genes in the baculovirus genome, namely chiA (chitinase) [41,42] and cath (cathepsin) [43,44], which are not essential for virus growth in cell culture. Finally, other approaches to improve baculovirus vectors involve the generation of non-lytic BEVS by random mutagenesis of viral genomes and the inclusion of foreign genes (*vankyrin* genes) from the insect *Campoletis sonorensis* ichnovirus (for review see [4] and [45]).

Recently, a baculovirus vector expression cassette, denominated TB, containing rearranged baculovirus-derived genetic regulatory elements acting in cascade has been described [4]. This novel cassette conferred significant production improvements to the BEVS, including prolonged cell integrity after infection, improved protein integrity, and up to a 4-fold increase in recombinant protein production yields in insect cells with respect to a standard baculovirus vector. The expression cassette consists of a cDNA encoding the baculovirus transactivation factors IE1 and IE0, expressed under the control of the *polh* promoter, and a homologous repeated transcription enhancer sequence operatively cis-linked to *p10* chimeric promoters. The prolonged cell integrity observed in cells infected by baculoviruses harboring the novel expression cassette reduced the characteristic proteolysis and aberrant forms frequently found in baculovirus-derived recombinant proteins [4].

Since the TB expression cassette was tested only with a model protein, here we studied its use for the production of VLPs. The selected antigens corresponded to the well characterized PCV2-Cap and RHDV-VP60 antigens. The first antigen is protective when expressed by a recombinant baculovirus [46–48] and constitutes the vaccines marketed by two pharmaceutical companies (Porcilis^®^ PCV from Merck and CircoFLEX^®^ from Boehringer Ingelheim). The RHDV-VP60 protein-based VLPs vaccine is not yet commercialized, mainly because of the associated production costs; however, several publications have demonstrated its protective effect in experimental vaccination experiments [17,22,26,49].

The TB expression cassette significantly increased (around 300%) the expression of both PCV2-Cap and RHDV-VP60 antigens in *Sf9* cells grown and infected in suspension with respect to a standard baculovirus (*polh* promoter). This increase in production did not modify the size or shape of the VLPs, as shown by EM (Figs 4 and 5). The kinetics of the expression of the two recombinant proteins was also modified by the TB(+) baculoviruses, which expressed the recombinant proteins earlier than a standard baculovirus under the control of the *polh* promoter. Also, the production was prolonged in infected cells for at least 24 h. This prolonged productivity correlated with a greater cell viability in cells infected by the TB(+) baculoviruses with respect to the standard viruses (data not shown). This observation thus confirms the previous observations with baculoviruses modified by this expression cassette [4]. In addition, in the case of RHDV-VP60 expressed by the TB(-) baculovirus, some minor reactive bands with the specific monoclonal antibody were observed. These bands were not observed in the extracts of cells infected by the TB(+) baculovirus. This finding suggests that the TB(+) baculovirus prevented the synthesis and accumulation of RHDV-VP60 protein forms that were not correctly processed in the infected cells. A similar observation was made with the purified RHDV VLPs, where a single Coomassie blue-stained band was detected when using the TB(+) virus, and several bands of higher and lower MW were observable in the VLPs produced by the TB(-) baculovirus.

In conclusion, here we have demonstrated that the TB expression cassette significantly increases the efficiency of the BEVS for VLP production. This novel cassette has the potential to considerably reduce the costs of subunit vaccine production, particularly those based on VLPs. The use of TB(+) baculovirus vectors provides a broad-based strategy for production of subunit human and animal vaccines, especially when the use of such biological products is restrained by production costs.
